# Supplementary material for: Integrated Analysis of the Lung Microbiome and Metabolome Reveals Associations Between Amino Acid Metabolism and Pulmonary Fibrosis in a Bleomycin-Induced Mouse Model
Source: Int J Mol Sci. 2026 Jun 30;27(13):5895. doi: 10.3390/ijms27135895 (PMC13362081; doi:10.3390/ijms27135895)
Supplement: Supplementary file 1 [file ijms-27-05895-s001.zip › result/5.MetKeggEnrichment/B_28d.vs.C_28d/B_28d.vs.C_28d_all.KEGG_Enrich.scatterplot.pdf]

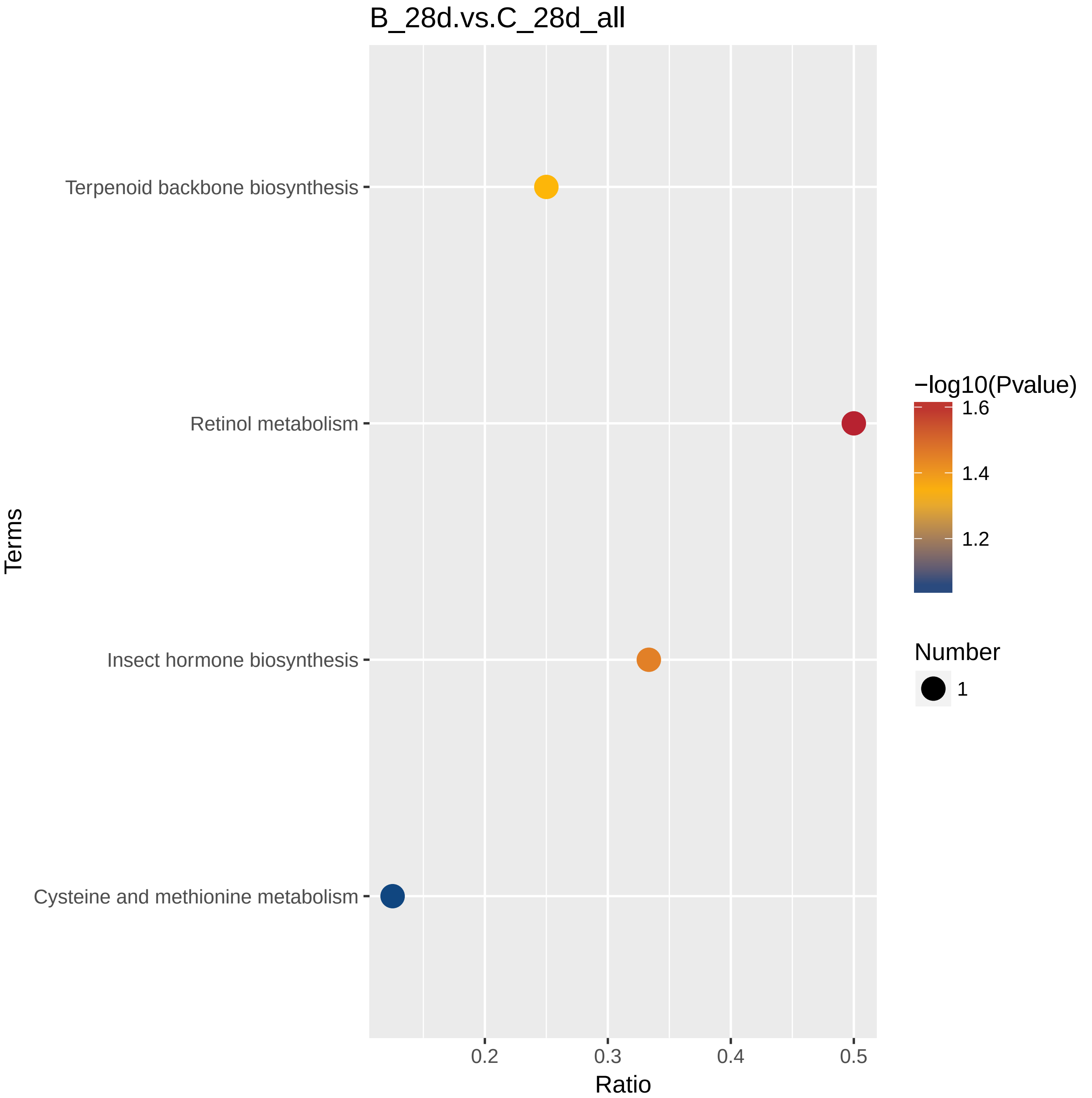

B_28d.vs.C_28d_all
Terpenoid backbone biosynthesis
Retinol metabolism
Insect hormone biosynthesis
Cysteine and methionine metabolism
Terms
0.2
0.3
0.4
0.5
Ratio
−log10(Pvalue)
1.6
1.4
1.2
Number
1
